# Lessons learned from a muscle study in nail-patella syndrome

**DOI:** 10.1186/s13023-025-03911-0

**Published:** 2025-07-28

**Authors:** Luisa Paul, Anne Schänzer, Christel Depienne, Andreas Hentschel, Nicolai Kohlschmidt, Ulrike Schara-Schmidt, Christopher Jannik Nelke, Andreas Roos, Heike Kölbel

**Affiliations:** 1 Department of Pediatric Neurology, Centre for Neuromuscular Disorders, Centre for Translational Neuro- and Behavioral Sciences, University Hospital Essen, Essen, Germany; 2 Department of Pediatric Cardiology and Congenital Heart Diseases, Centre for Child and Adolescent Medicine, Heidelberg University Medical Center, Heidelberg, Germany; 3 Institute of Neuropathology, Justus-Liebig-University Giessen, Giessen, Germany; 4 Institute of Human Genetics, University Hospital Essen, University Duisburg-Essen, Essen, Germany; 5 Leibniz Institute für Analytische Wissenschaften – ISAS – e.V., Dortmund, Germany; 6 Institute of Clinical Genetics and Tumour Genetics, Bonn, Germany; 7 National Centre of Genetics, Dudelange, Luxemburg; 8 Institute of Neurology, University Hospital Düsseldorf, Heinrich Heine University Düsseldorf, Düsseldorf, Germany; 9 Children’s Hospital of Eastern Ontario Research Institute, University of Ottawa, Ottawa, ON K1H 8L1 Canada

**Keywords:** Nail-patella syndrome, Elbow contractures, Emery dreyfuss muscle dystrophy, LMX1B, Muscle proteomics

## Abstract

**Background:**

Nail-patella (NPS) syndrome is an autosomal dominant disorder caused by mutations in the *LMX1B* gene and manifests with involvement of kidneys, nails, eyes as well as skeletal musculature. NPS shows some clinical similarities with Emery-Dreifuss muscular dystrophy. However, thus far human muscle tissue has not been analysed in the context of NPS to precisely clarify the muscular involvement in this multi-systemic disease.

**Methods:**

To study the effects of a missense variant in *LMX1B* on human skeletal muscle, histological, immunofluorescence and ultra-structural studies were performed on a deltoid muscle biopsy performed at the age of 2 aiming to analyse potential pathologies in muscle fibres in addition to unbiased proteomic profiling to identify dysregulated proteins.

**Results:**

Microscopic work-up of the muscle biopsy revealed no striking pathologies, except for some atrophic fibres. The proteomic analyses unveiled a clustered number of dysregulated keratin proteins among the downregulated proteins.

**Conclusion:**

Although NPS can also present with a muscular phenotype indicated by muscular weakness of the upper extremities, elevated CK levels and contractures of the elbow joint, there is no evidence of primary muscular involvement due to expression of mutant LMX1B. The examination of human skeletal muscle tissue confirmed the findings from the animal models showing that the skeletal muscle symptoms of NPS may be the result of a developmental disorder of the extremities that leads to impaired muscle mobilisation.

**Supplementary Information:**

The online version contains supplementary material available at 10.1186/s13023-025-03911-0.

## Introduction

The *LMX1B* gene (MIM 602575), located on chromosome 9q33.3, encodes LIM homeobox transcription factor 1-beta, an 379 amino acid spanning protein resident in the nucleus [1]. LMX1B is expressed in different tissues during early embryonic development and is important for the dorso-ventral pattering of soft tissue of the limb, the nails, the patella and the long bones during skeletal development. Acting as a transcription factor, LMX1B hereby modulates differentiation of the eyes and development of neuronal populations in the CNS as well as early morphogenesis of the glomerular basement membrane [2]. Heterozygous loss-of-function variants of *LMX1B* cause nail-patella syndrome (NPS; MIM: #161,200). Disease causative variants can be either missense, nonsense or frame-shift mutations, and are most frequent localized within the homedomain motive of the protein [3, 4]. The incidence of NPS is approximately 1:50.000 [5].

NPS is moreover characterized by malformations of the skeletal system including patella hypoplasia (75%), iliac horns (with exostosis [68%]), elbow abnormalities with dysplasia of the radial head, hypoplasia of the lateral epicondyle and capitellum and prominence of the medial epicondyle (33%), of nails with dysplasia of the nails (98%), triangular lunulae (88%), split nails as well as ocular (glaucoma [9.6%], ocular hypotension [7.2%]) and renal symptoms (nephropathy [48%]) [6]. Additional symptoms include impaired hearing (45.8%), neurological manifestations affecting the peripheral nervous system (numbness, tingling or burning sensation of the distal limb [25%]) as well as the central nervous system (epilepsy [6%]) and dental problems (crumbling teeth [23%]) [2].

Although studies on skeletal muscle are existing for mouse models of the disease, thus far there is no study investigating the impact of heterozygous LMX1B variants on skeletal muscle in human. Previous studies have shown that Lmx1b activity in skeletal progenitor cells is required for the formation of the dorsal pattern of limbs, but patterning of the skeletal and connective tissues can also be uncoupled, suggesting a degree of autonomy in the formation of the musculoskeletal system [7]. Muscle progenitor cells are naïve and depend on signals from the surrounding tissue to form specific arrangements of muscle fibres and eventually a stereotyped adult form [8, 9]. In contrast, skeletal progenitor cells appear to have some autonomy, as the limb skeleton develops in a normal pattern in the absence of muscle. *Lmx1b* expression was found to be localised in the dorsal joint-forming regions, in developing tendons and ligaments, but was not detectable in migrating myocytes [7]. Studies during joint, tendon and muscle formation in *Lmx1b* knockout (KO) mice revealed that *Lmx1b* activity is required in both proximal and distal limb bud tissues, with *Lmx1b* function apparently being more critical in distal tissues. Hence, *Lmx1b* is expressed in tendon and skeletal progenitor cells of the dorsal limb bud in chick and mice and in progenitor cells of muscle connective tissue [10, 11]. Consequently, Lmx1b’ role in muscle patterning and development seems to be indirect, likely mediated by the connective tissue [12].

Here we describe the clinical findings in a paediatric patient with a heterozygous pathogenic variant in *LMX1B*, suffering from NPS symptoms presenting with an Emery-Dreifuss muscular dystrophy-like phenotype. Furthermore, we report on microscopic and proteinogenic studies performed on a deltoideus muscle biopsy of this patients, thus introducing the first muscle study in *LMX1B*-patients, including, histology, electron microscopy, and proteomic profiling aiming to gain more insights into the potential biochemical characteristics of *LMX1B*-mutant muscle.

## Methods

### Patient

Our index introduced in this study was phenotyped in the Department of Pediatric Neurology of the University Hospital of Essen (Duisburg-Essen University). Written informed consent for clinical description, genetic studies, and utilization of the muscle biopsy specimen for research purposes was obtained from the patients’ parents. The study was conducted according to the guidelines of the Declaration of Helsinki and approved by the Ethics Committee of University of Duisburg-Essen (19-9011-BO) in the context of the NMD-GPS project (https://nmd-gps.net/).

### Microscopic studies of muscle biopsy

Deltoideus muscle biopsy investigated in this study has initially been collected for diagnostic purposes including histology, enzyme histochemistry, immunofluorescence and immunohistochemical investigations. Serial cryosections (7 µm) of transversely-oriented muscle blocks were stained according to standard procedures with hematoxylin and eosin (H&E), Gomori trichrome (GT), oil red, COX-SDH and SDH, ATP-ases at pH 4.3 and 9.4 and nicotinamide adenine dinucleotide tetrazolium reductase (NADH-TR). Immunofluorescence studies focussed on Caveolin 3, Dystrophin (1/2/3), α-/β-/γ-/δ- Dystroglycan, Laminin α2, Emerin, Dysferlin, Collagen IV as well as Telethoin, Calpain and Dysferlin, LMX1b (Millipore) and LMX1B (GeneTex). Secondary antibodies were conjugated with Alexa 488 (donkey anti-rabbit IgG antibody; Molecular Probes). Nuclei were visualized with 4′,6-diamidino-2-phenylindol dihydrochloride (DAPI; Carl Roth, Karlsruhe, Germany). Hereby, we included muscle tissues without any pathological features upon microscopic evaluation as normal disease control (or physiological internal control) for all reactions. Microscopy was performed using a Zeiss Axioplan epifluorescence microscope and a Zeiss Axio Cam ICc 1.

Glutaraldehyde-fixed specimens were processed for ultrastructural examination by standard procedures. Small samples were fixed with 6% glutaraldehyde/0.4 M PBS at RT for 24 h, washed 5 times in 0.1 M Epon-PBS and processed with a tissue processor with 1% osmium tetroxide (Leica EM TP). Samples were dehydrated by immersion in increasing ethanol concentrations (25%, 35%, 50%, 70%, 75%, 85%, 100%), and after that infiltrated with a propylene oxide and a propylene oxide/resin mixture (Agar 100 Resin Kit). Polymerization of the resin was accomplished at 60 °C for 24 h. Semi-thin Sects. (990 nm thickness) were prepared and stained with 1% Richardson to assess overall tissue morphology. Ultrathin Sects. (190 nm thickness) were prepared and placed at 200 mesh copper grids (3.05 mm). Samples were examined and photographed using a with a transmission electron Zeiss microscope, type EM 109 using with a 2 K-CCD-Camera from TRS.

### Genetic analysis

DNA samples from the index patient and his parents were subjected to whole exome sequencing. Libraries were created with an Illumina exome cpture (38 Mb target) kit and sequenced with a mean target coverage of 80 × (Genomics Platform, Broad Institute of MIT and Harvard, Cambridge, USA). Data analysis was carried out in two stages, first searching for pathogenic/likely pathogenic variants in genes known to be associated with the clinical presentation (in silico panel). If no variants were identified, the full exome was interrogated by applying a stringent criterion searching for homozygous highly damaging variants (i.e., nonsense, splice region, and frame-shift) absent in the control population (gnomAD; http://gnomad.broadinstitute.org).

### Proteomic profiling

Muscle tissue of our index patient was lysed in 200 μl of 50 mM Tris–HCl (pH 7.8) buffer, 5% SDS and cOmplete ULTRA protease inhibitor (Roche) using a Bioruptor® sonication device (Diagenode) for 10 min (30 s on, 30 s off, 10 cycles) at 4 °C. To ensure complete lysis, an additional sonication step was performed by ultrasound (30 s, 1 s/1 s, amplitude 40%), followed by centrifugation at 4 °C and 20,000 g for 15 min. Protein concentration of the supernatant was determined by BCA assay (Pierce) according to the manufacturer’s protocol. Disulfide bonds were reduced by addition of 10 mM Tris(2-carboxyethyl)phosphine hydrochloride (TCEP) at 37 °C for 30 min, and free sulfhydryl bonds were alkylated with 15 mM IAA at room temperature (RT) in the dark for 30 min. From each sample 100 μg of protein was used for proteolysis utilizing the S-trap protocol (Protifi) and a protein-to-trypsin ratio of 20:1. The samples were incubate for 2 h at 37 °C. Proteolysis was stopped by acidification of the sample (pH < 3.0) with formic acid to acidify the sample.

All proteolytic digests were checked for complete digestion after desalting by monolithic column separation (PepSwift monolithic PS-DVB PL-CAP200-PM, Dionex) on an inert Ultimate 3000 HPLC (Dionex, Germering, Germany) by direct injection of 1 μg protein per sample. A binary gradient (solvent A: 0.1% TFA, solvent B: 0.08% TFA, 84% ACN) of 5–12% B in 5 min and then of 12–50% B in 15 min was applied at a flow rate of 2.2 μl/min and at 60 °C. UV traces were recorded at 214 nm. For proteomic profiling, three control samples derived from male children of Caucasian origin (10 months, 12 months, and 14 months of age, respectively) with an unremarkable family history were included. In these control biopsies histological and laboratory investigations remained unremarkable [13].

### Transcriptomic profiling

To investigate *LMX1B* expression in human skeletal muscle, RNA Sequencing was performed on 8 skeletal muscle samples stored in liquid nitrogen before utilization. Briefly, mRNAs were first enriched with oligo (dT) beads. The enriched mRNAs were fragmented according to the manufacturer’s instructions. First and second-strand cDNAs were subsequently synthesised. The cDNA fragments were end-repaired and adenylated at 3′ ends, and universal adapters were ligated to the cDNA fragments, followed by index addition and library enrichment by limited-cycle PCR. The sequencing libraries were validated using an NGS Kit on the Agilent 5300 Fragment Analyser (Agilent Technologies, Palo Alto, CA, USA), and quantified using a Qubit 4.0 Fluorometer (Invitrogen, Carlsbad, CA, USA). The sequencing libraries were multiplexed and loaded onto the flow cell on an Illumina NovaSeq 6000 instrument, according to the manufacturer’s instructions. The samples were sequenced using a 2 × 150 Pair-End (PE) configuration v1.5. Image analysis and base calling were conducted on a NovaSeq instrument using the NovaSeq Control Software v1.7 on the NovaSeq instrument. The raw sequence data (.bcl files) generated by the Illumina NovaSeq were converted into fastq files and de-multiplexed using the Illumina bcl2fastq programme version 2.20. One mismatch was allowed for the index sequence identification. To understand whether *LXM1B* is expressed in skeletal muscle tissue, we reanalysed our previously published dataset. We quantified the expression of *LXM1B, COL1A2* and *TTN*, as previously described, in non-diseased muscle control samples [14]. The transcriptomic data has been deposited under Gene Expression Omnibus (GEO) GSE260786.

## Results

### Clinical findings

The patient is the second child of healthy, non-consanguineous parents, who first presented at the age of 15 month in our neuropediatric outpatient clinic.

After an uneventful pregnancy and normal birth, two urinary tract infections in the second and fourth month of life occurred. A renal ultrasound was conducted and a duplex kidney with an enlarged kidney pelvis (right side) was diagnosed. A double kidney, also known as a double collecting system, is a congenital malformation in which, in the mildest form, only the renal pelvis is divided into two parts and, in the most severe form, one kidney has developed two complete urinary tracts. This is a relatively common variation of the urinary tract, occurring in about 1% of the population. Further renal examinations showed mild dilated ureter and mild disorders in the renal function (protein-crea-ratio).

The parents reported a motor developmental delay, showing crawling at 13 months of age, to stand up at 15 months of age, and to walk independently at 22 months of age. Particularly in the first year of life, proximal muscular weakness in the upper arms and shoulder girdle was noticeable, which made it impossible for the child to crawl up. During physiotherapy, very mild contractures in the elbow joint were noticed for the first time around 10 months of age. The cognitive development and language development were unremarkable.

Clinical examinations at the age of 15 months in our department of pediatric neurology (University Medicine Essen, Germany) revealed joint contractures of both elbows/ saddle thumb joint, muscular hypotonia of the trunk and atrophic muscles of the upper extremities with muscular weakness and macrocephaly. The elbow contractures are more limiting to function than weakness. Laboratory analysis presented mild elevated CK-level of max. 244 U/l (normal value < 180 U/l). At the age of 24 month, MRI of cranium, spine and shoulder girdle muscles including the upper arm muscles showed no abnormalities (data not shown). Due to the EDMD-like phenotype, developmental delay and mild elevated CK-level a muscle biopsy was performed for diagnostic purposes. At this time, the genetic diagnosis of NPS had not been made. Certainly, a muscle biopsy is not necessary to confirm an NPS.

**Fig. 1:**
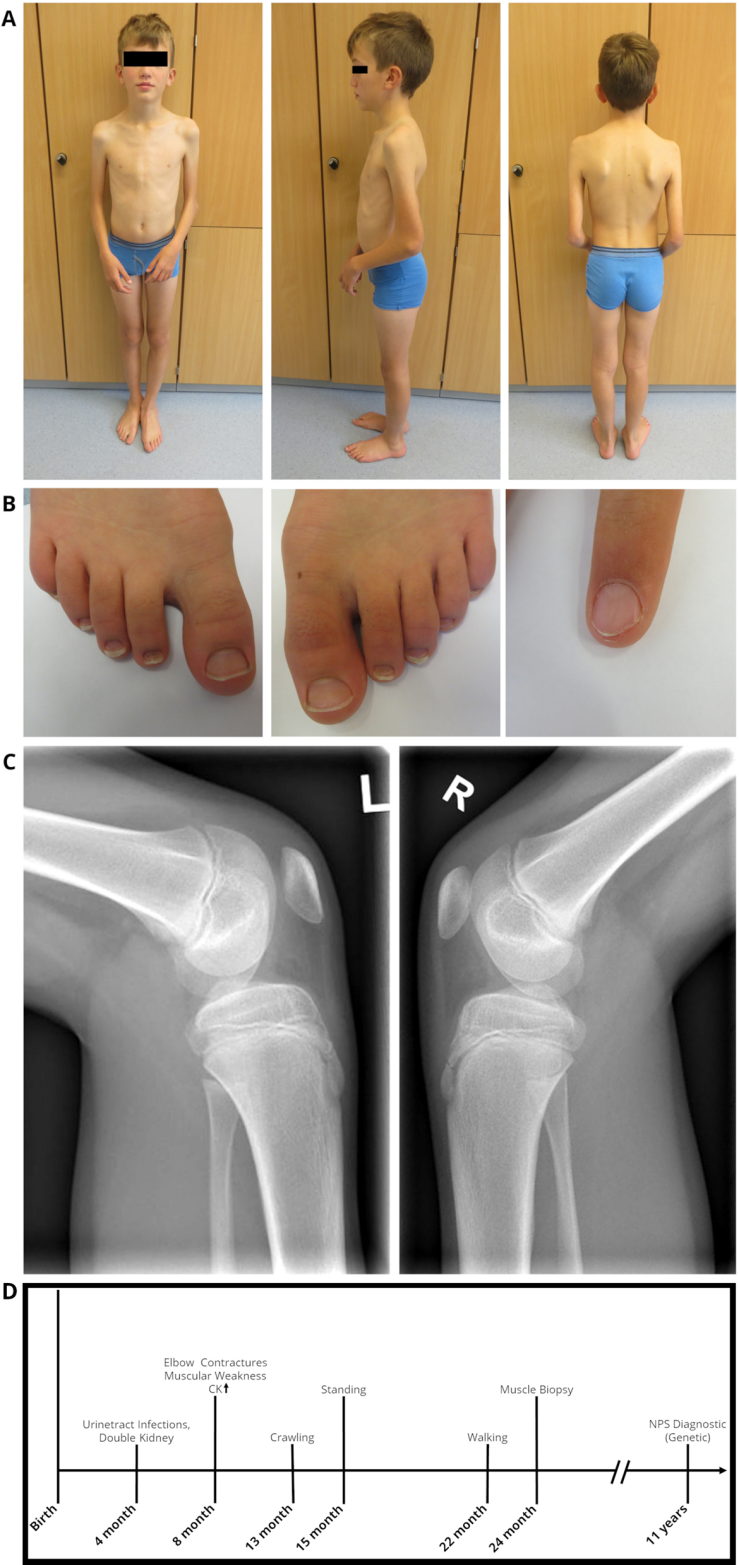
Clinical findings in our index. At the age of 12 years: **A** Pronounced elbow contractures on both sides, with atrophy of the upper arm muscles and scapulae alatae, **B** mild dystrophic nails of the feet and triangular lunula of the finger nail, **C** Patellar hypoplasia on both sides. **D** Schematic representation of the diagnostic work-up along with clinical findings in our NPS patient

Further neuropediatric follow-up examinations revealed constant muscle weakness of the upper extremities, scapulae alatae and mild motor developmental delays with myalgias (Fig. 1A). The index finger of the left hand showed a triangular lunula but no dystrophic signs (Fig. 1B). Physiotherapy and ergotherapy occur as supporting therapy—mild improvements of the contractures have been shown.

Currently, at the age of 16, the patient is part of a football team and shows no muscle weakness, although the contractures of the elbow are more pronounced than at the time of the muscle biopsy.

### Muscle biopsy findings

**Fig. 2:**
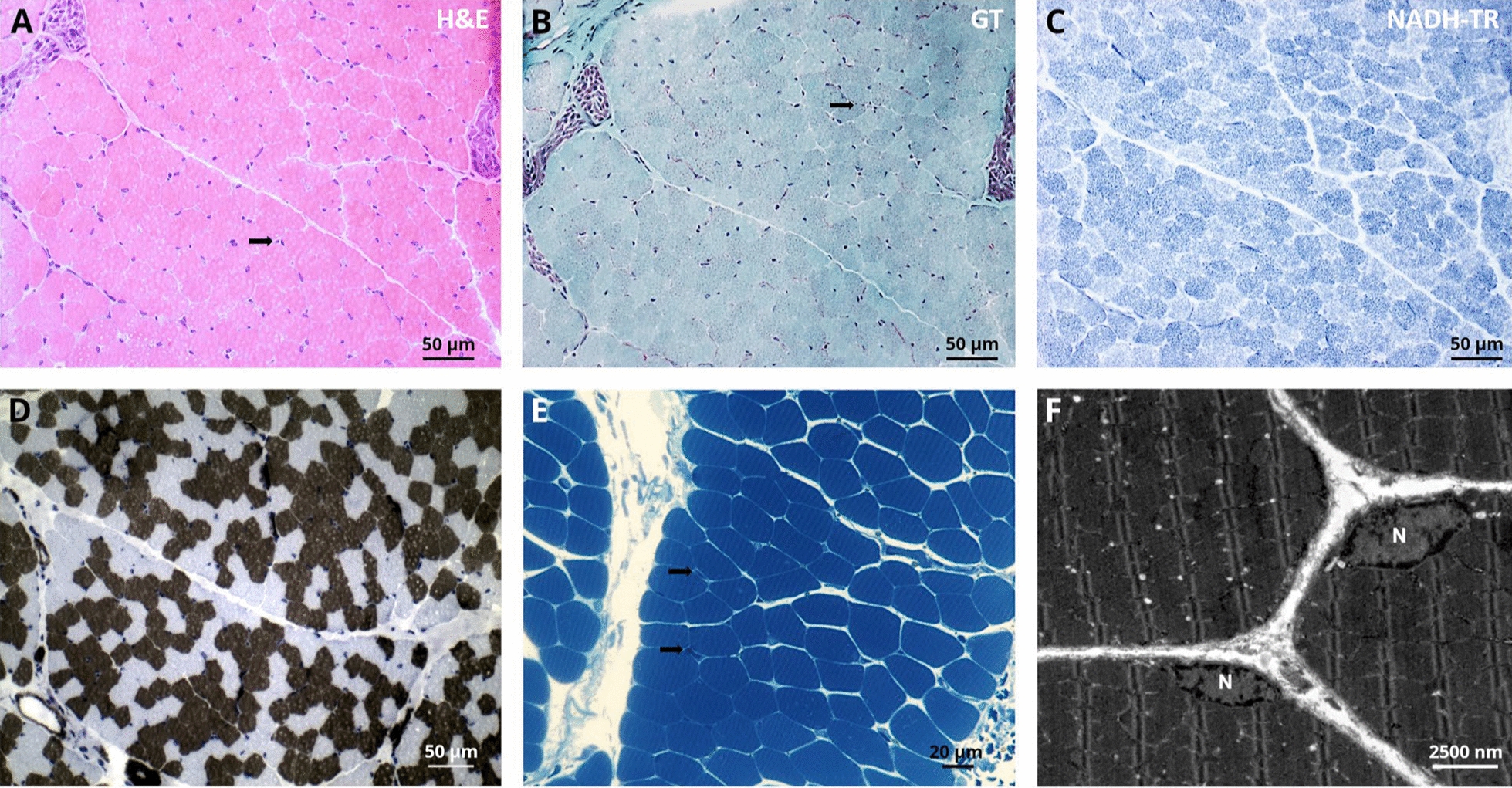
Histopathological findings in deltoid muscle of our NPS patient. Light microscopic findings on *LMX1B* mutant muscle, H&E stains and semi-thin sections show normal morphology with some atrophic fibres (arrow)(A + E). Gomori trichrome (GT) and nicotinamide adenine dinucleotide tetrazolium reductase (NADH-TR) show normal morphology. ATPase stain (pH 4.3) shows moderate variation of fibre calibres **D**. Ultrastructural analysis revealed marginal nuclei (N) normal in shape and structure including integrity of the nuclear envelope. The sarcomere architecture is regular without specific alterations **F**

Since the patient developed symptoms mainly in the upper extremity resembling a EDMD-like phenotype, a biopsy of the deltoid muscle was collected for further diagnostic work-up at the age of 2 years and histology showed a skeletal musculature with very mild abnormalities defined by discrete atrophic fibres. The following routine stains were carried out on the basis of clinical suspicion of congenital myopathy, and an immunoblot analysis was performed for suspected EDMD. Protein abundance and distribution for Caveolin 3, Dystrophin (1/2/3), α-/β-/γ-/δ- Dystroglycan, Laminin α2, Emerin, Dysferlin, Collagen IV as well as Telethoin, Calpain, Dysferlin and LMX1B studied by immunofluorescence appeared normal (data not shown). Results of immunoblot of Emerin were unremarkable (data not shown). Microscopic studies of semi-thin sections (Fig. 2B) as well as ultra-structural investigations were performed to further elucidate potential morphological perturbations. Here, resin sections revealed only a moderate variation of fibre calibres (Fig. 2D). Ultrastructural analysis revealed marginal nuclei normal in shape and structure including integrity of the nuclear envelope. The sarcomere architecture was regular without specific alterations (Fig. 2F).

### Molecular genetic findings

Genetic diagnostic testing of the *LMNA* and *COL6A1-3* genes revealed no pathological findings. Variants in *ITGA7* gene (c.8246G > A; p.(Arg275His)) and *RYR1* gene (c.2648C > T; p.(Ala882Val)) of uncertain significance were found. However, as those were also not fitting with the phenotype observed in our patient, the patient was included in a research project (NMD-GPS; https://nmd-gps.net/) for exome sequencing unveiling a heterozygous missense variant in *LMX1B* (c.668G > A; p.(Arg223Gln)) in exon 4 (https://www.ncbi.nlm.nih.gov/clinvar/RCV000007422/). Sanger-based segregation studies confirmed a de novo origin of the variant.

### Proteomic Profiling

**Fig. 3:**
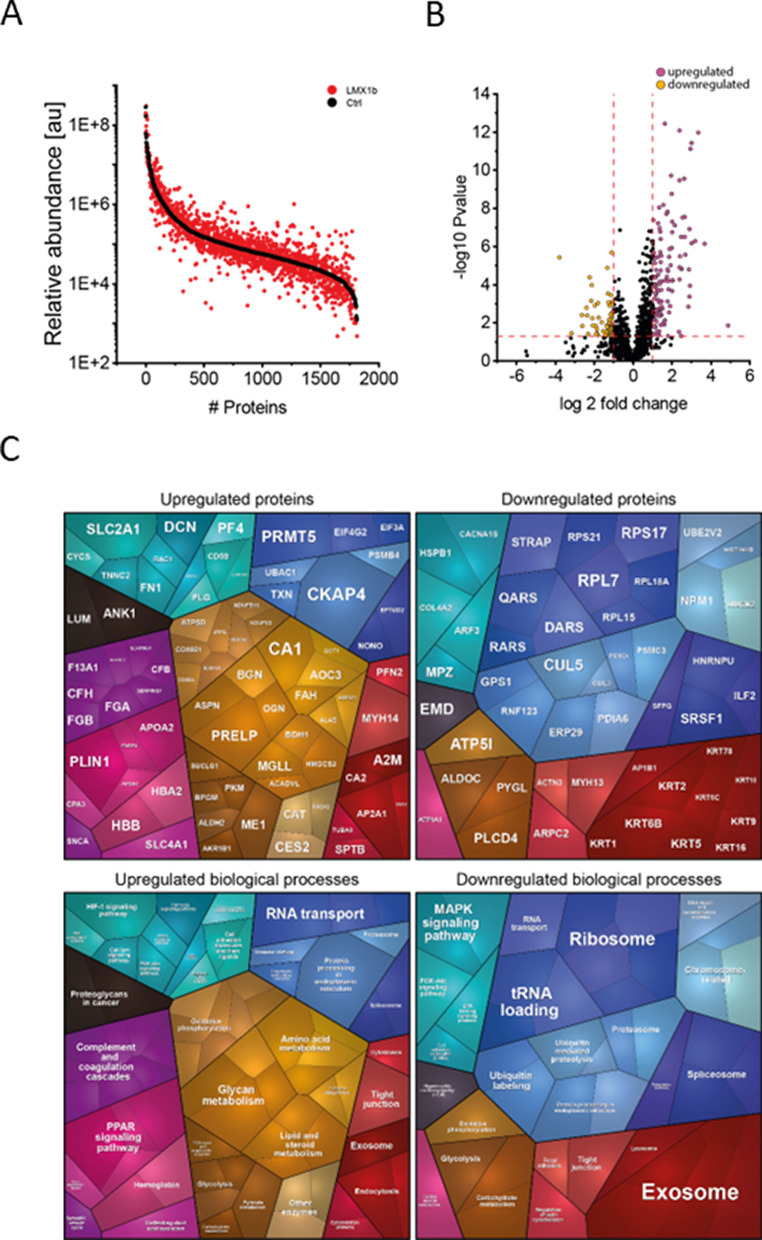
Proteomic findings in deltoid muscle of our NPS patient. **A** Abundance plot showing the dynamic range of all proteins identified in proteins extracts of deltoid muscle via liquid chromatography coupled to tandem mass spectrometry based on their relative quantification using always the 3 highest abundant peptides for each protein, allowing protein comparison within an experiment. All identified proteins of the controls (black) are sorted with decreasing abundance while the patient (red) was plotted in the same order to directly compare the different abundances. All identified proteins cover a dynamic range of eight orders of magnitude. **B** Volcano plot highlighting statistically significant increased proteins (purple dots) as well as decreased proteins (yellow dots). **C** Proteomaps-based in silico analysis of cellular functions affected by the respective dysregulated proteins. Dysregulated proteins are shown in the upper panel separated for upregulated proteins (left) and downregulated proteins (right). Affected biological processes are shown in the lower panel accordingly

To analyse the potential impact on the biochemical integrity of skeletal muscle, unbiased proteomic profiling was conducted in a data-independent-acquisition mode. This analytical approach allowed the quantification of proteins spanning eight orders of magnitude (Fig. 3A) and identified 128 upregulated and 64 downregulated proteins (Fig. 3B). A proteomaps-based pathway analysis was conducted for increased and decreased proteins separately: Upregulated proteins impact on various biological processes including activation of the complement system, metabolism, RNA-transport as well as exosome function and endocytosis. Downregulated proteins also impact on exosome function in addition to ribosomal function, tRNA-loading, MAPK signaling, and protein ubiquitination among others (Fig. 3C).

Among the 192 differential abundant proteins, 16 of the up-regulated and 26 of the downregulated proteins are localized in the nucleus (Table A1). Of note, 59 proteins are associated with diseases and some proteins are associated with diseases that phenotypically overlap with the spectrum of clinical presentation seen in NPS (Table 2). These disease associations of dysregulated proteins for instance include lipodystrophy (PLN1), deafness (MYH14) and peripheral neuropathy with myopathy, hoarseness and hearing loss (MYH14). GTR1 is associated with neurologic disorders and GLUT1 deficiency syndrome is clinically characterized by encephalopathy, delayed development, microcephaly, motor incoordination, and spasticity, Epilepsy, dystonia and other neurological defects syndrome.

### Transcriptomic profiling

**Fig. 4:**
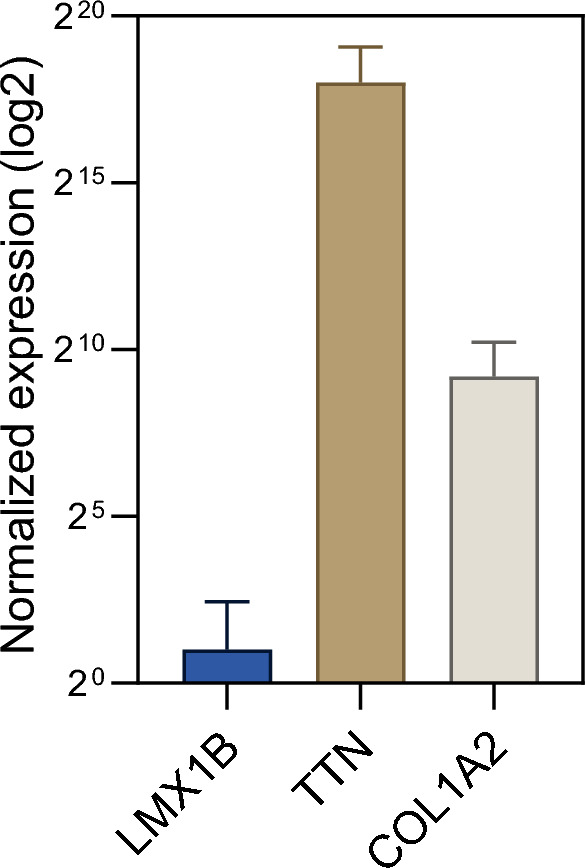
Transcript studies toward expression analysis of LMX1B in human muscle. Bulk RNA-sequencing of eight non-diseased controls. We compared the expression of *LXM1B* to genes with known expression in skeletal muscle (*COL1A2* and *TTN*). *LMX1B* is not expressed in muscle tissue

Finally, we aimed to address the unmet question if *LXM1B* is expressed in human skeletal muscle. For this purpose, we utilized a transcriptomic dataset consisting of bulk RNA-sequencing of eight non-diseased controls. We compared the expression of *LXM1B* to genes with known expression in skeletal muscle, including C*OL1A2* and *TTN*. Here, we observed that *LMX1B* is not expressed in human muscle cells (Fig. 4).

## Discussion

Due to the clinical and laboratory findings, we assumed at first the diagnosis of Emery-Dreifuss muscular dystrophy (EDMD), a rare disease presenting with a wide age range of clinical onset. Of note first EDMD symptoms are contractures before muscular weakness occurs, appearing especially with humeroperoneal distribution [15]. Our NPS patient presented at the beginning of the disease with a muscular phenotype including elbow contractures, muscular hypotonia of the upper extremity, mild CK elevation and proximal weakness in the upper limbs resembling a phenotype with overlaps to EDMD. However, a novel missense mutation (p.(Arg223Gln)) in the transcription factor LMX1B was identified as being causative for NPS. A detailed examination of the myopathology was performed to elucidate the potential vulnerability of skeletal muscle in the cause of NPS. Microscopic studies showed very mild pathologies in terms of atrophic fibres. This finding might most likely be explained by the existing inactivity of the upper extremity due to the elbow contractures [16].

Analysis of the proteomic signature in deltoid muscle showed multiple dysregulated proteins that were associated with several diseases for example central nervous system (CNS) disorders, deafness and polyneuropathy. It is well known, that Lmx1b is essential for development of certain neuron populations in the CNS [5] and three functional Lmx1b alleles are required in zebrafish for correct numbers of excitatory spinal interneurons at later developmental stages [17]. Some of the clinical features of NPS patients fit to these findings like such as Parkinsonism, hearing problems or sensory disturbances in the distal limbs [6]. We also identified a clustered number of dysregulated keratin proteins among the downregulated proteins impacting on exosome function according to proteomaps-based prediction of affected biological processes. Of note, keratins play an important role in epithelial cells and tissue (for example nails) [18]. In this context it is important to note that KRT2, KRT5, KRT16 and KRT9 are downregulated between 15 and 40% and localized both in the sarcolemma and in the nucleus. KRT2 plays an important role in the epidermis of the skin (cornification, keratinocyte activation and proliferation) and is important for the epidermal barrier [19]. KRT5 is responsible for the development of the cytoskeleton of the basal keratins and stabilizes the skin [20]. KRT16 is an important checkpoint of the immune system of the skin barrier. Mutations in KRT16 are associated with hypertrophic nail dystrophy [21]. KRT9 is important for the structure of the skin in keratin filament assembly. Mutations are associated with thickening of the skin [22]. It is important to emphasize that, although the described dysregulation of keratins is highly unlikely to be directly linked to pathophysiological cascades in the examined tissue—likely due to their lower functional relevance in muscle—, the identified dysregulation of keratins could, in contrast, be of pathophysiological relevance for the involvement of the nails. Clinically, our patient suffers from mild dystrophic nails of the feet with triangular lunula, which are typical findings in a milder phenotype of NPS [23].

The molecular function of Lmx1b in embryonic development has been investigated in detail in numerous studies on animal models (mice and chicken). With regard to muscle development, it was demonstrated that Lmx1b also controls the organization and shape of the dorsal musculature, although it is not expressed in the muscle itself (Fig. 4). Muscle precursors are not committed to a specific pattern [9, 24] and when muscle precursors migrate into the dorsal connective tissue, they remain Lmx1b-negative, although they are invested in Lmx1b-positive epimysium [7]. Therefore, the role of Lmx1b in muscle formation and development is indirect and is likely mediated by the connective tissue [12]. In accordance with literature knowledge, no LMX1B protein could be detected, neither in the muscle biopsy of our patient nor in control biopsies, again strengthening the concept of an impact of altered connective tissue on development of contractures in the etiology of NPS in our patient. We can confirm this assumption with our extended investigation of human muscle tissue of a patient with NPS. It revealed no evidence of a muscular dystrophic or myopathic process, nor did it show any impairment of the myonuclear structures, including the nuclear envelope, although some of the dysregulated proteins are also localized in the nucleus. We can assume that the mild dysregulation of the proteins is not sufficient to lead to structural changes in the muscle cell, as we know from EDMD, for example. One might rather assume that the protein dysregulations unveiled by unbiased mass spectrometry-based proteomics represent cellular attempts of compensation of LMX1B mutation and/ or represent general dysregulation which only have an impact in cellular populations vulnerable in NPS. Especially the identified changes in abundances of nuclear proteins (without pathology of myonuclei on the ultra-structural level) might accord with this hypothesis.

To summarize, phenotypic similarities between our patient and the clinical presentation of Emery-Dreifuss disease with contractures of the elbows, muscular hypotonia and mild elevated CK prompted us to intensively characterize the muscle biopsy of our NPS patient on both, the morphological and biochemical level. Hence, this is the first detailed description of consequences of LMX1B mutation in human skeletal muscle. However, apart from some atrophic fibres, there was no evidence of pronounced primary skeletal muscle involvement in NPS. This in turn emphasizes that the primary function of LMX1B might be restricted to the development of the limbs. Thus, the skeletal muscle symptoms of NPS might be the result of a developmental disorder of the extremities leading to perturbed muscle mobilization. Overall, our analyses did not reveal any autonomic muscle pathology. In this context, the biochemical changes unraveled by unbiased proteomics may (partially) occur from this immobilization and could also represent activation of compensatory cascades toward restoration of muscle cell integrity, an aspect demonstrated by our ultra-structural findings.

## Limitations

Unfortunately, we were unable to recruit any further muscle biopsies from NPS patients, so that all analyses originate from one patient, and we also lack analyses of other tissues such as skin or nerve tissue.

## Supplementary Information

Additional file 1.

Additional file 2.

## Data Availability

All data generated or analysed during this study are included in this published article and its supplementary information files.

## Abbreviations

CNS: Central Nervous System
EDMD: Emery Dreyfuss Muscular Dystrophy
KRT: Keratin
LMX1B: LIM Homeobox Transcription Factor 1 Beta
NPS: Nail Patella Syndrome
OAE: Otoacoustic Emissions
SHG: Second Harmonic Generation
CK: Creatine Kinase
