# Supplementary material for: Lessons learned from a muscle study in nail-patella syndrome
Source: Orphanet J Rare Dis. 2025 Jul 28;20:384. doi: 10.1186/s13023-025-03911-0 (PMC12306095; doi:10.1186/s13023-025-03911-0)
Supplement: Supplementary file 1 — Additional file 1. [file 13023_2025_3911_MOESM1_ESM.docx]

**Table A1:**

**Proteomic analysis**: Dysregulated proteins within patient’s muscle biopsy showing nucleus localization.

| **PG.Protein**  **Accessions** | **Gene** | **PG. Protein names** | **Protein** | **Protein function** | **Localisation (nucleus)** | **NME 10/Ctrl** | **pValue** |
| --- | --- | --- | --- | --- | --- | --- | --- |
| O14744 | PRMT5 | ANM5 | Protein arginine N-methyltransferase 5 | Arginine methyltransferase that can both catalyze the formation of omega-N monomethylarginine (MMA) and symmetrical dimethylarginine (sDMA), with a preference for the formation of MMA | nucleus | 8,04 | 0,0000 |
| O60936 | NOL3 | NOL3 | Nucleolar protein 3 | Isoform 1: May be involved in RNA splicing.Isoform 2: Functions as an apoptosis repressor that blocks multiple modes of cell death. | nucleolus | 7,44 | 0,0000 |
| Q15029 | EFTUD2 | U5S1 | 116 kDa U5 small nuclear ribonucleoprotein component | Required for pre-mRNA splicing as component of the spliceosome, including pre-catalytic, catalytic and post-catalytic spliceosomal complexes | nucleus | 4,08 | 0,000 |
| P06703 | S100A6 | S10A6 | Protein S100-A6 | May function as calcium sensor and modulator, contributing to cellular calcium  signaling. | nucleus envelope | 3,58 | 0,000 |
| P15090 | FABP4 | FABP4 | Fatty acid-binding protein, adipocyte | Lipid transport protein in adipocytes. | nucleus | 2,76 | 0,000 |
| P37840 | SNCA | SYUA | Alpha-synuclein | Neuronal protein that plays several roles in synaptic activity such as regulation of  synaptic vesicle trafficking and subsequent neurotransmitter release. | nucleus | 2,64 | 0,003 |
| Q15233 | NONO | NONO | Non-POU domain-containing octamer-binding protein | DNA- and RNA binding protein, involved in several nuclear processes. | nucleus, nucleolus,  nucleus speckle | 2,46 | 0,001 |
| P28070 | PSMB4 | PSB4 | Proteasome subunit beta type-4 | Non-catalytic component of the 20S core proteasome complex involved in the proteolytic degradation of most intracellular proteins. | nucleus | 2,42 | 0,043 |
| P17931 | LGALS3 | LEG3 | Galectin-3 | Galactose-specific lectin which binds IgE. | nucelus | 2,35 | 0,002 |
| P10599 | TXN | THIO | Thioredoxin | Participates in various redox reactions through the reversible oxidation of its active center dithiol to a disulfide and catalyzes dithiol-disulfide exchange reactions. | nucleus | 2,23 | 0,000 |
| Q9NRG7 | SDR39U1 | D39U1 | Epimerase family protein SDR39U1 | Putative NADP-dependent oxidoreductase. | nucleus | 2,14 | 0,016 |
| P10909 | CLU | CLUS | Clusterin | Isoform 1: Functions as extracellular chaperone that prevents aggregation of non native proteins , Isoform 6: Does not affect caspase or BAX-mediated intrinsic apoptosis and TNF-induced NF-kappa-B-activity. Isoform 4: Does not affect caspase or BAX-mediated intrinsic apoptosis and TNF-induced NF-kappa-B-activity. | nucleus | 2,13 | 0,043 |
| P26447 | S100A4 | S10A4 | Protein S100-A4 | Calcium-binding protein that plays a role in various cellular processes including motility, angiogenesis, cell differentiation, apoptosis, and autophagy. | nucleus | 2,05 | 0,002 |
| P14618 | PKM | KPYM | Pyruvate kinase PKM | Glycolytic enzyme that catalyzes the transfer of a phosphoryl group from phosphoenolpyruvate (PEP) to ADP, generating ATP. | nucleus | 2,03 | 0,000 |
| P49773 | HINT1 | HINT1 | Histidine triad nucleotide-binding protein 1 | Exhibits adenosine 5'-monophosphoramidase activity, hydrolyzing purine nucleotide phosphoramidates with a single phosphate group such as adenosine 5'monophosphoramidate (AMP-NH2) to yield AMP and NH2 | nucleus | 2,00 | 0,001 |
| P13716 | ALAD | HEM2 | Delta-aminolevulinic acid dehydratase | Catalyzes an early step in the biosynthesis of tetrapyrroles. | nucleus | 2,00 | 0,005 |
| P17980 | PSMC3 | PRS6A | 26S proteasome regulatory subunit 6A | Component of the 26S proteasome, a multiprotein complex involved in the ATP-dependent degradation of ubiquitinated proteins. | nucleus | 0,49 | 0,003 |
| P18124 | RPL7 | RL7 | 60S ribosomal protein L7 | Component of the large ribosomal subunit. | nucleus, nucleolus | 0,49 | 0,020 |
| Q9Y3F4 | STRAP | STRAP | Serine-threonine kinase receptor-associated protein | The SMN complex catalyzes the assembly of small nuclear ribonucleoproteins (snRNPs), the building blocks of the spliceosome, and thereby plays an important role in the splicing of cellular pre-mRNAs. | nucleus | 0,49 | 0,023 |
| Q07955 | SRSF1 | SRSF1 | Serine/arginine-rich splicing factor 1 | Plays a role in preventing exon skipping, ensuring the accuracy of splicing and  regulating alternative splicing. Interacts with other spliceosomal components, via the RS domains, to form a bridge between the 5'- and 3'-splice site binding components, U1 snRNP and U2AF. | nucleus speckle | 0,49 | 0,042 |
| Q9UHP9 | SMPX | SMPX | Small muscular protein | Plays a role in the regulatory network through which muscle cells coordinate their structural and functional states during growth, adaptation, and repair. | nucleus | 0,48 | 0,001 |
| Q1KMD3 | HNRNPUL2 | HNRL2 | Heterogeneous nuclear ribonucleoprotein U-like protein 2 | RNA binding. | nucleus | 0,48 | 0,005 |
| P06748 | NPM1 | NPM | Nucleophosmin | Involved in diverse cellular processes such as ribosome biogenesis, centrosome duplication, protein chaperoning, histone assembly, cell proliferation, and regulation of tumor suppressors p53/TP53 and ARF. | nucleolus, nucleoplasm | 0,47 | 0,003 |
| P04792 | HSPB1 | HSPB1 | Heat shock protein beta-1 | Small heat shock protein which functions as a molecular chaperone probably maintaining denatured proteins in a folding-competent state. | nucleus | 0,47 | 0,005 |
| P08708 | RPS17 | RS17 | 40S ribosomal protein S17 | RNA binding , structural constituent of ribosome, cytoplasmic translation, erythrocyte homeostasis, ribosomal small subunit biogenesis, rRNA processing, translation, translational initiation | nucleoplasm | 0,46 | 0,019 |
| Q5VWP3 | MLIP | MLIP | Muscular LMNA-interacting protein | Required for precocious cardiac adaptation to stress through integrated regulation of the AKT/mTOR pathways and FOXO1. | nucleus, nucleus envelope, PML body | 0,46 | 0,000 |
| Q00839 | HNRNPU | HNRPU | Heterogeneous nuclear ribonucleoprotein U | DNA- and RNA-binding protein involved in several cellular processes such as  nuclear chromatin organization, telomere-length regulation, transcription, mRNA alternative splicing and stability, Xist-mediated transcriptional silencing and mitotic cell progression | nucleus, nucleus matrix,  nucleus speckle | 0,46 | 0,012 |
| P26583 | HMGB2 | HMGB2 | High mobility group protein B2 | Multifunctional protein with various roles in different cellular compartments. | nucleus | 0,46 | 0,036 |
| P23246 | SFPQ | SFPQ | Splicing factor, proline- and glutamine-rich | DNA- and RNA binding protein, involved in several nuclear processes. | nucleus speckle, nucleus matrix | 0,45 | 0,004 |
| Q9BRC7 | PLCD4 | PLCD4 | 1-phosphatidylinositol 4,5-bisphosphate phosphodiesterase delta-4 | Hydrolyzes the phosphatidylinositol 4,5-bisphosphate (PIP2) to generate 2 second messenger molecules diacylglycerol (DAG) and inositol 1,4,5-trisphosphate (IP3). | nucleus | 0,42 | 0,016 |
| O15144 | ARPC2 | ARPC2 | Actin-related protein 2/3 complex subunit 2 | Actin-binding component of the Arp2/3 complex, a multiprotein complex that  mediates actin polymerization upon stimulation by nucleation-promoting factor (NPF) | nucleus | 0,41 | 0,019 |
| P35908 | KRT2 | K22E | Keratin, type II cytoskeletal 2 epidermal | Probably contributes to terminal cornification | nucleus | 0,41 | 0,037 |
| Q15819 | UBE2V2 | UB2V2 | Ubiquitin-conjugating enzyme E2 variant 2 | Has no ubiquitin ligase activity on its own. The UBE2V2/UBE2N  heterodimer catalyzes the synthesis of non-canonical poly-ubiquitin chains that are linked through 'Lys-63'. | nucleoplasm, nucleus | 0,41 | 0,048 |
| P13647 | KRT5 | K2C5 | Keratin, type II cytoskeletal 5 | There are two types of cytoskeletal and microfibrillar keratin: I (acidic; 40-55 kDa) and II (neutral to basic; 56-70 kDa). | nucleus | 0,29 | 0,006 |
| Q12905 | ILF2 | ILF2 | Interleukin enhancer-binding factor 2 | Appears to function predominantly as a heterodimeric complex with ILF3. | nucleolus, nucleus | 0,29 | 0,044 |
| Q13098 | GPS1 | CSN1 | COP9 signalosome complex subunit 1 | ssential component of the COP9 signalosome complex (CSN), a complex involved in various cellular and developmental processes. | nucleus | 0,28 | 0,038 |
| P16401 | HIST1H1B | H15 | Histone H1.5 | Histone H1 protein binds to linker DNA between nucleosomes forming the macromolecular structure known as the chromatin fiber. | nucleus | 0,24 | 0,015 |
| P43686 | PSMC4 | PRS6B | 26S proteasome regulatory subunit 6B | Component of the 26S proteasome, a multiprotein complex involved in the ATP-dependent degradation of ubiquitinated proteins. | nucleus | 0,24 | 0,006 |
| P08779 | KRT16 | K1C16 | Keratin, type I cytoskeletal 16 | Epidermis-specific type I keratin that plays a key role in skin. Acts as a regulator of innate immunity in response to skin barrier breach: required for some inflammatory checkpoint for the skin barrier maintenance. | nucleus | 0,20 | 0,004 |
| O43598 | DNPH1 | DNPH1 | 2'-deoxynucleoside 5'-phosphate N-hydrolase 1 | Catalyzes the cleavage of the N-glycosidic bond of deoxyribonucleoside 5'-monophosphates to yield deoxyribose 5-phosphate and a purine or pyrimidine base. Deoxyribonucleoside 5'-monophosphates containing purine bases are preferred to those containing pyrimidine bases. | nucleus | 0,19 | 0,002 |
| P35527 | KRT9 | K1C9 | Keratin, type I cytoskeletal 9 | May serve an important special function either in the mature palmar and plantar skin tissue or in the morphogenetic program of the formation of these tissues. Plays a role in keratin filament assembly. | nucleus | 0,16 | 0,004 |
| Q13618 | CUL3 | CUL3 | Cullin-3 | Core component of multiple cullin-RING-based BCR (BTB-CUL3-RBX1) E3 ubiquitin-protein ligase complexes which mediate the ubiquitination and subsequent proteasomal degradation of target proteins. BCR complexes and ARIH1 collaborate in tandem to mediate ubiquitination of target proteins | nucleus | 0,11 | 0,038 |

Green =upregulated proteins, red = downregulated proteins.
