# Supplementary material for: Lessons learned from a muscle study in nail-patella syndrome
Source: Orphanet J Rare Dis. 2025 Jul 28;20:384. doi: 10.1186/s13023-025-03911-0 (PMC12306095; doi:10.1186/s13023-025-03911-0)
Supplement: Supplementary file 2 — Additional file 2. [file 13023_2025_3911_MOESM2_ESM.docx]

**Table A2:**

**Proteomic analyses:** Dysregulated proteins and their associated diseases presenting with phenotypical overlap to Nail patella syndrome.

| **PG.Protein**  **Accessions** | **Gene** | **Protein** | **PG. Protein names** | **Protein function** | **Localisation** | **NME 10/Ctrl** | **p-Value** | **Associated Diseases (#OMIM)** |
| --- | --- | --- | --- | --- | --- | --- | --- | --- |
| O60240 | PLIN1 | Perilipin-1 | PLN1 | Modulator of adipocyte lipid metabolism. | endoplasmatic reticulum, lipid droplet | 8,05 | 0,000 | # 613877 LIPODYSTROPHY, FAMILIAL PARTIAL, TYPE 4; FPLD4 |
| Q7Z406 | MYH14 | Myosin-14 | MYH14 | Cellular myosin that appears to play  a role in cytokinesis, cell shape, and specialized  functions such as secretion and capping. | cytoskeleton, cytosol, extracellular region,  other locations (brush border, growth cone, membrane, myosin II filament) | 7,60 | 0,000 | # 600652 DEAFNESS, AUTOSOMAL DOMINANT 4A; DFNA4A  # 614369 PERIPHERAL NEUROPATHY, MYOPATHY, HOARSENESS, AND HEARING LOSS; PNMHH |
| P11166 | SLC2A1 | Solute carrier family 2,  facilitated glucose transporter member 1 | GTR1 | Facilitative glucose transporter,  which is responsible for constitutive or basal glucose uptake | plasma membrane, others (melanosome, photoreceptor  inner segment) | 7,46 | 0,000 | # 606777 GLUT1 DEFICIENCY SYNDROME 1; GLUT1DS1  # 612126 GLUT1 DEFICIENCY SYNDROME 2; GLUT1DS2,  # 614847 EPILEPSY, IDIOPATHIC GENERALIZED, SUSCEPTIBILITY TO, 12; EIG12   # 601042 DYSTONIA 9; DYT9  # 608885 STOMATIN-DEFICIENT CRYOHYDROCYTOSIS WITH NEUROLOGIC DEFECTS; SDCHCN |
| O60936 | NOL3 | Nucleolar protein 3 | NOL3 | Isoform 1: May be involved in RNA splicing.  Isoform 2:Functions as an apoptosis repressor that blocks multiple modes of cell death. | isoform 1:nucleus, isoform 3: cytoplasma,  cytosol. Isoform 2: mitochondrion, cytoplasm, cytosol, others | 7,44 | 0,000 | # 614937 MYOCLONUS, FAMILIAL, 1; MYOCL1 |
| P16157 | ANK1 | Ankyrin-1 | ANK1 | Attaches integral membrane proteins to cytoskeletal elements; binds to the erythrocyte membrane protein band 4.2, to Na-K ATPase, to the lymphocyte membrane protein GP85, and to the cytoskeletal proteins fodrin, tubulin, vimentin and desmin.  Isoform Mu17: Together with obscurin in skeletal muscle may provide a molecular link between the sarcoplasmic reticulum and myofibrils. | isoform Er1: cytoskeleton,  isoform Mu17: membrane, m line. Isoform Mu18+19+20: sarcoplasmic reticulum | 6,45 | 0,000 | # 182900 SPHEROCYTOSIS, TYPE 1; SPH1 |
| P01857 | IGHG1 | Immunoglobulin heavy  constant gamma 1 | IGHG1 | Constant region of immunoglobulin  heavy chains | Extracellular region, plasma membrane | 5,94 | 0,000 | # 254500 MYELOMA, MULTIPLE |
| P02730 | SLC4A1 | Band 3 anion transport  protein | B3AT | Functions both as a transporter that  mediates electroneutral anion exchange across the cell membrane and as a structural protein. | plasma membrane | 5,68 | 0,000 | # 166900 OVALOCYTOSIS, SOUTHEAST ASIAN; SAO  # 612653 SPHEROCYTOSIS, TYPE 4; SPH4  # 179800 RENAL TUBULAR ACIDOSIS, DISTAL, 1; DRTA1  # 611590 RENAL TUBULAR ACIDOSIS, DISTAL, 4, WITH HEMOLYTIC ANEMIA; DRTA4 # 185020 CRYOHYDROCYTOSIS; CHC |
| P01871 | IGHM | Immunoglobulin heavy  constant mu | IGHM | Constant region of immunoglobulin  heavy chains. | isoform 1:extracellular region,  isoform 2: plasma membrane | 5,62 | 0,000 | # 601495 AGAMMAGLOBULINEMIA 1, AUTOSOMAL RECESSIVE; AGM1 |
| P69905 | HBA1 | Hemoglobin subunit  alpha | HBA | Involved in oxygen transport  from the lung to the various peripheral tissues. | cytosol, extracellular region (blood microparticel, extracelluar exosome/ region /space),  other locations (endocytic vesicle lumen, haptoglobin-hemoglobin complex) | 5,53 | 0,000 | # 140700 HEINZ BODY ANEMIAS,   # 604131ALPHA-THALASSEMIA,   # 613978 HEMOGLOBIN H DISEASE; HBH |
| P68871 | HBB | Hemoglobin subunit  beta | HBB | Involved in oxygen transport  from the lung to the various peripheral tissues. | cytosol, extracellular region (blood microparticel, extracelluar exosome/ region /space),  other locations (endocytic vesicle lumen, ficolin-1-rich granule lumen, haptoglobin-hemoglobin complex, tertiary granule lumen) | 5,31 | 0,000 | # 140700 HEINZ BODY ANEMIAS  # 613985 BETA-THALASSEMIA  # 603903 SICKLE CELL ANEMIA  # 603902 BETA-THALASSEMIA, DOMINANT INCLUSION BODY TYPE |
| Q9BXN1 | ASPN | Asporin | ASPN | Negatively regulates periodontal ligament (PDL) differentiation and mineralization to ensure that the PDL is not ossified and to maintain homeostasis of the tooth-supporting system. | extracelluar region (extracellular matrix) | 4,48 | 0,000 | # 607850 OSTEOARTHRITIS SUSCEPTIBILITY 3; OS3,   # 603932 INTERVERTEBRAL DISC DISEASE; IDD |
| P54868 | HMGCS2 | Hydroxymethylglutaryl-CoA  synthase, mitochondrial | HMCS2 | Catalyzes the first irreversible step in ketogenesis, condensing acetyl-CoA  to acetoacetyl-CoA to form HMG-CoA, which is converted by HMG-CoA reductase (HMGCR) into mevalonate. | mitochondrion | 4,14 | 0,000 | # 605911 3-HYDROXY-3-METHYLGLUTARYL-CoA SYNTHASE-2 DEFICIENCY; HMGCS2D |
| Q15029 | EFTUD2 | 116 kDa U5 small nuclear  ribonucleoprotein component | U5S1 | Required for pre-mRNA splicing as component  of the spliceosome, including pre-catalytic, catalytic and post-catalytic spliceosomal complexes | nucleus | 4,08 | 0,000 | # 610536 MANDIBULOFACIAL DYSOSTOSIS, GUION-ALMEIDA TYPE; MFDGA |
| P01876 | IGHA1 | Immunoglobulin heavy constant  alpha 1 | IGHA1 | Constant region of immunoglobulin heavy chains. Immunoglobulins, also known as antibodies,  are membrane-bound or secreted glycoproteins produced by B lymphocytes. | plasma membrane (cell membrane),  extracellular region (secreted) | 4,01 | 0,000 | # 254500 MYELOMA, MULTIPLE |
| P07585 | DCN | Decorin | PGS2 | May affect the rate of fibrils  formation. | extracellular matrix | 3,96 | 0,000 | # 610048 CORNEAL DYSTROPHY, CONGENITAL STROMAL; CSCD |
| P21810 | BGN | Biglycan | PGS1 | May be involved in collagen fiber assembly. | extracellular matrix | 3,85 | 0,000 | # 300989 MEESTER-LOEYS SYNDROME; MRLS  # 300106 SPONDYLOEPIMETAPHYSEAL DYSPLASIA, X-LINKED; SEMDX |
| O75208 | COQ9 | Ubiquinone biosynthesis  protein COQ9, mitochondrial | COQ9 | Lipid-binding protein involved in the biosynthesis  of coenzyme Q, also named ubiquinone, an essential lipid-soluble electron transporter for aerobic cellular respiration. | mitochondrion | 3,71 | 0,000 | # 614654 COENZYME Q10 DEFICIENCY, PRIMARY, 5; COQ10D5 |
| P00488 | F13A1 | Coagulation factor XIII  A chain | F13A | Factor XIII is activated by thrombin and calcium ion to a transglutaminase that catalyzes the formation of gamma-glutamyl-epsilon-lysine cross-links between fibrin chains, thus stabilizing the fibrin clot. | extracellular region (secreted), cytoplasm | 3,67 | 0,003 | # 613225 FACTOR XIII, A SUBUNIT, DEFICIENCY OF |
| Q0ZGT2 | NEXN | Nexilin | NEXN | Involved in regulating cell migration  through association with the actin cytoskeleton. | cytoskeleton, adheren junction, Z line | 3,48 | 0,050 | # 613122 CARDIOMYOPATHY, DILATED, # 613876 CARDIOMYOPATHY, FAMILIAL HYPERTROPHIC, 20; CMH20 1CC; CMD1CC |
| P53597 | SUCLG1 | Succinate--CoA ligase [ADP/GDP-forming]  subunit alpha, mitochondrial | SUCA | Succinyl-CoA synthetase functions  in the citric acid cycle (TCA), coupling the hydrolysis of succinyl-CoA to the synthesis of either ATP or GTP and thus represents the only step of substrate-level phosphorylation in the TCA. | mitochondrion | 3,48 | 0,000 | # 245400 MITOCHONDRIAL DNA DEPLETION SYNDROME 9 (ENCEPHALOMYOPATHIC TYPE WITH METHYLMALONIC ACIDURIA); MTDPS9 |
| P01834 | IGKC | Immunoglobulin kappa constant | IGKC | Constant region of immunoglobulin light chains. | cell membrane, extracellular region (secreted) | 3,40 | 0,000 | # 614102 IMMUNOGLOBULIN KAPPA LIGHT CHAIN DEFICIENCY; IGKCD |
| P02671 | FGA | Fibrinogen alpha chain | FIBA | Cleaved by the protease thrombin  to yield monomers which, together with fibrinogen beta (FGB) and fibrinogen gamma (FGG), polymerize to form an insoluble fibrin matrix. | extracellular (secreted) | 3,16 | 0,008 | # 202400 AFIBRINOGENEMIA, CONGENITAL   # 105200 AMYLOIDOSIS, FAMILIAL VISCERAL   # 616004 DYSFIBRINOGENEMIA, CONGENITAL |
| P16452 | EPB42 | Erythrocyte membrane  protein band 4.2 | EPB42 | Probably plays an important role  in the regulation of erythrocyte shape and mechanical properties. | cell membrane, lipid-anchor, cytoplasmic side,  cytoskeleton | 3,13 | 0,000 | # 612690 SPHEROCYTOSIS, TYPE 5; SPH5 |
| P02675 | FGB | Fibrinogen beta chain | FIBB | Cleaved by the protease thrombin  to yield monomers which, together with fibrinogen alpha (FGA) and fibrinogen gamma (FGG), polymerize to form an insoluble fibrin matrix. | extracellular region (secreted) | 3,07 | 0,013 | # 202400 AFIBRINOGENEMIA, CONGENITAL  # 616004 DYSFIBRINOGENEMIA, CONGENITAL |
| P02751 | FN1 | Fibronectin | FINC | Fibronectins bind cell surfaces and various compounds including collagen, fibrin, heparin, DNA, and actin | extracellular matrix | 2,93 | 0,012 | # 601894 GLOMERULOPATHY WITH FIBRONECTIN DEPOSITS 2; GFND2  # 184255 SPONDYLOMETAPHYSEAL DYSPLASIA, CORNER FRACTURE TYPE; SMDCF |
| Q12805 | EFEMP1 | EGF-containing  fibulin-like extracellular matrix  protein 1 | FBLN3 | Binds EGFR, the EGF  receptor, inducing EGFR autophosphorylation and the activation of downstream signaling pathways. | extracellular space, extracellular matrix | 2,79 | 0,001 | # 126600 DOYNE HONEYCOMB RETINAL DYSTROPHY; DHRD |
| P13987 | CD59 | CD59 glycoprotein | CD59 | Potent inhibitor of the complement  membrane attack complex (MAC) action. | extracellular region (secreted),  cellmembrane, GPI-anchor | 2,77 | 0,004 | # 612300 HEMOLYTIC ANEMIA, CD59-MEDIATED, WITH OR WITHOUT IMMUNE-MEDIATED POLYNEUROPATHY; HACD59 |
| P02787 | TF | Serotransferrin | TRFE | Transferrins are iron binding  transport proteins which can bind two Fe^3+^ ions in association with the binding of an anion, usually bicarbonate. | extracellular region (secreted) | 2,73 | 0,000 | # 209300 ATRANSFERRINEMIA |
| P99999 | CYCS | Cytochrome c | CYC | Electron carrier protein. | mitochondrion intermembrane space | 2,68 | 0,000 | # 612004 THROMBOCYTOPENIA 4; THC4 |
| P30049 | ATP5F1D | ATP synthase subunit  delta, mitochondrial | ATPD | Mitochondrial membrane ATP  synthase (F_1_F_0_ ATP synthase or Complex V) produces ATP from ADP in the presence of a proton gradient across the membrane which is generated by electron transport complexes of the respiratory  chain. | mitochondrion, mitochondrion inner membrane | 2,65 | 0,000 | # 618120 MITOCHONDRIAL COMPLEX V (ATP SYNTHASE) DEFICIENCY,  NUCLEAR TYPE 5; MC5DN5 |
| P37840 | SNCA | Alpha-synuclein | SYUA | Neuronal protein that plays several roles in synaptic activity such as regulation of synaptic vesicle trafficking and subsequent  neurotransmitter release. | cytoplasm, nucleus, extracellular region  (secreted), membrane, synapse | 2,64 | 0,003 | # 168601 PARKINSON DISEASE 1, AUTOSOMAL DOMINANT; PARK1,  # 605543 PARKINSON DISEASE 4, AUTOSOMAL DOMINANT; PARK4,   # 127750 DEMENTIA, LEWY BODY; DLB |
| P00738 | HP | Haptoglobin | HPT | As a result of hemolysis, hemoglobin  is found to accumulate in the kidney and is secreted in the urine. | extracelluar region (secreted) | 2,62 | 0,002 | # 614081 ANHAPTOGLOBINEMIA; AHP |
| P63000 | RAC1 | Ras-related C3 botulinum  toxin substrate 1 | RAC1 | Plasma membrane-associated small GTPase which  cycles between active GTP-bound and inactive  GDP-bound states. | cytoplasm, cell membrane, lipid-anchor, cytoplasmic side, melanosome,  lamellipodium, dendrite, synapse | 2,62 | 0,000 | # 617751 MENTAL RETARDATION, AUTOSOMAL DOMINANT 48; MRD48 |
| P02768 | ALB | Serum albumin | ALBU | Binds water, Ca^2+^, Na^+^, K^+^, fatty acids,  hormones, bilirubin and drugs (Probable). | extracellular region (secreted) | 2,62 | 0,000 | # 615999 HYPERTHYROXINEMIA, FAMILIAL DYSALBUMINEMIC; FDAH,  # 616000 ANALBUMINEMIA; ANALBA |
| P11277 | SPTB | Spectrin beta chain,  erythrocytic | SPTB1 | Spectrin is the major constituent of the cytoskeletal  network underlying the erythrocyte plasma  membrane. | cytoskeleton, cell cortex | 2,58 | 0,000 | # 617948 ELLIPTOCYTOSIS 3; EL3,  # 616649 SPHEROCYTOSIS, TYPE 2; SPH2 |
| P04040 | CAT | Catalase | CATA | Occurs in almost all aerobically respiring  organisms and serves to protect cells from the toxic effects of hydrogen peroxide. | Peroxisome | 2,56 | 0,005 | # 614097 ACATALASEMIA |
| P02647 | APOA1 | Apolipoprotein A-I | APOA1 | Participates in the reverse transport of cholesterol  from tissues to the liver for excretion by promoting cholesterol efflux from tissues and by acting as a cofactor for the lecithin cholesterol acyltransferase (LCAT). | extracelluar region (secreted) | 2,53 | 0,002 | # 618463 HYPOALPHALIPOPROTEINEMIA, PRIMARY, 2,   # 105200 AMYLOIDOSIS, FAMILIAL VISCERAL |
| P05546 | SERPIND1 | Heparin cofactor 2 | HEP2 | Thrombin inhibitor activated by the glycosaminoglycans,  heparin or dermatan sulfate. | endoplasmatic reticulum lumen, extracellular exosome,  extracellular region, extracellular space | 2,51 | 0,002 | # 612356 HEPARIN COFACTOR II DEFICIENCY |
| Q15233 | NONO | Non-POU domain-containing  octamer-binding protein | NONO | DNA- and RNA binding protein, involved in several  nuclear processes. | nucleus, nucleolus, nucleus speckle,  chromosome | 2,46 | 0,001 | # 300967 INTELLECTUAL DEVELOPMENTAL DISORDER, X-LINKED, SYNDROMIC 34; MRXS34 |
| Q96RL7 | VPS13A | Vacuolar protein sorting-associated  protein 13A | VP13A | Required for the formation or stabilization of ER-mitochondria  contact sites which enable transfer of lipids between the ER and mitochondria | endoplasmatic reticulum membrane, peripheral membrane protein,  golgi apparatus, endosome membrane protein, mitochondrion outer membrane , mitochondrion outer membrane, lysosome membrane, lipid droplets, neuronal dense core vesicle | 2,46 | 0,000 | # 200150 CHOREOACANTHOCYTOSIS; CHAC |
| P28070 | PSMB4 | Proteasome subunit  beta type-4 | PSB4 | Non-catalytic component of the 20S core proteasome  complex involved in the proteolytic degradation of most intracellular proteins. | cytoplasm, nucleus | 2,42 | 0,043 | # 617591 PROTEASOME-ASSOCIATED AUTOINFLAMMATORY SYNDROME 3; PRAAS3 |
| P16930 | FAH | Fumarylacetoacetase | FAAA | Catalytic activity, protein binding, involved in step 6  of the subpathway that synthesizes acetoacetate and fumarate from L-phenylalanine | cytosol, extracellular exosome | 2,33 | 0,004 | # 276700 TYROSINEMIA, TYPE I; TYRSN1 |
| P07738 | BPGM | Bisphosphoglycerate  mutase | PMGE | Plays a major role in regulating hemoglobin oxygen  affinity by controlling the levels of its allosteric effector 2,3-bisphosphoglycerate (2,3-BPG). | cytosol, extracellular exosome | 2,31 | 0,008 | # 222800 ERYTHROCYTOSIS, FAMILIAL, 8; ECYT8 |
| P00751 | CFB | Complement factor B | CFAB | Factor B which is part of the alternate pathway  of the complement system is cleaved by factor D into 2 fragments: Ba and Bb. Bb, a serine protease, then combines with complement factor 3b to generate the C3 or C5 convertase. | extracellular region (secreted) | 2,26 | 0,000 | # 615489 MACULAR DEGENERATION, AGE-RELATED, 14; ARMD14,  # 612924 HEMOLYTIC UREMIC SYNDROME, ATYPICAL, SUSCEPTIBILITY TO, 4; AHUS4,  # 615561 COMPLEMENT FACTOR B DEFICIENCY; CFBD |
| P00918 | CA2 | Carbonic anhydrase 2 | CAH2 | Essential for bone resorption and osteoclast  differentiation (By similarity). | cytoplasm, cell membrane | 2,23 | 0,019 | # 259730 OSTEOPETROSIS, AUTOSOMAL RECESSIVE 3; OPTB3 |
| P05155 | SERPING1 | Plasma protease  C1 inhibitor | IC1 | Activation of the C1 complex is under control of the  C1-inhibitor. It forms a proteolytically inactive stoichiometric complex with the C1r or C1s proteases. May play a potentially crucial role in regulating important physiological pathways including complement activation, blood coagulation, fibrinolysis and the generation of kinins. | extracellular region (secreted) | 2,22 | 0,016 | # 106100 ANGIOEDEMA, HEREDITARY, 1; HAE1 |
| Q9NY65 | TUBA8 | Tubulin alpha-8 chain | TBA8 | Tubulin is the major constituent of microtubules. | cytoskeleton | 2,18 | 0,000 | # 609528 CEREBRAL DYSGENESIS, NEUROPATHY, ICHTHYOSIS, AND PALMOPLANTAR KERATODERMA SYNDROME |
| P20674 | COX5A | Cytochrome c oxidase subunit 5A, mitochondrial | COX5A | Component of the cytochrome c oxidase,  the last enzyme in the mitochondrial electron transport chain which drives oxidative phosphorylation. | mitochondrion inner membrane,  peripheral membrane protein, matrix side | 2,18 | 0,000 | # 619064 MITOCHONDRIAL COMPLEX IV DEFICIENCY, NUCLEAR TYPE 20; MC4DN20 |
| P08603 | CFH | Complement factor H | CFAH | Glycoprotein that plays an essential role  in maintaining a well-balanced immune response by modulating complement activation. | extracellular region (secreted) | 2,18 | 0,007 | # 126700 BASAL LAMINAR DRUSEN,  # 609814 COMPLEMENT FACTOR H DEFICIENCY; CFHD  # 235400 HEMOLYTIC UREMIC SYNDROME, ATYPICAL, SUSCEPTIBILITY TO, 1; AHUS1  # 610698 MACULAR DEGENERATION, AGE-RELATED, 4; ARMD4 |
| P00747 | PLG | Plasminogen | PLMN | Plasmin dissolves the fibrin of blood clots and acts as a proteolytic factor in a variety of other processes including embryonic development, tissue remodeling, tumor invasion, and inflammation. | extracellular region (secreted) | 2,17 | 0,004 | # 217090 PLASMINOGEN DEFICIENCY, TYPE I |
| P19404 | NDUFV2 | NADH dehydrogenase [ubiquinone]  flavoprotein 2, mitochondrial | NDUV2 | Core subunit of the mitochondrial membrane respiratory chain  NADH dehydrogenase (Complex I) which catalyzes electron transfer from NADH through the respiratory chain, using ubiquinone as an electron acceptor. | mitochondrion inner membrane, peripheral membrane protein,  matrix side | 2,12 | 0,000 | # 618229 MITOCHONDRIAL COMPLEX I DEFICIENCY, NUCLEAR TYPE 7; MC1DN7 |
| P49748 | ACADVL | Very long-chain specific acyl-CoA  dehydrogenase, mitochondrial | ACADV | Very long-chain specific acyl-CoA dehydrogenase  is one of the acyl-CoA dehydrogenases that catalyze the first step of mitochondrial fatty acid beta-oxidation, an aerobic process breaking down fatty acids into acetyl-CoA and allowing the production of energy from fats. | mitochondrion inner membrane,  peripheral membrane protein | 2,11 | 0,000 | # 201475 ACYL-CoA DEHYDROGENASE, VERY LONG-CHAIN, DEFICIENCY OF; ACADVLD |
| P01008 | SERPINC1 | Antithrombin-III | ANT3 | Most important serine protease inhibitor  in plasma that regulates the blood coagulation cascade. | extracellular space | 2,09 | 0,002 | # 613118 ANTITHROMBIN III DEFICIENCY; AT3D |
| P14854 | COX6B1 | Cytochrome c oxidase  subunit 6B1 | CX6B1 | Component of the cytochrome c oxidase, the last enzyme in the mitochondrial electron transport chain which  drives oxidative phosphorylation. | mitochondrion inner membrane,  peripheral membrane protein, intermembrane side | 2,05 | 0,000 | # 619051 MITOCHONDRIAL COMPLEX IV DEFICIENCY, NUCLEAR TYPE 7; MC4DN7 |
| P56556 | NDUFA6 | NADH dehydrogenase [ubiquinone] 1 alpha subcomplex subunit 6 | NDUA6 | Accessory subunit of the mitochondrial membrane respiratory  chain NADH dehydrogenase (Complex I), that is believed to be not involved in catalysis. | mitochondrion inner membrane,  peripheral membrane protein, matrix side | 2,03 | 0,000 | # 618253 MITOCHONDRIAL COMPLEX I DEFICIENCY, NUCLEAR TYPE 33; MC1DN33 |
| O95169 | NDUFB8 | NADH dehydrogenase [ubiquinone] 1 beta subcomplex subunit 8, mitochondrial | NDUB8 | Accessory subunit of the mitochondrial membrane  respiratory chain NADH dehydrogenase (Complex I), that is believed not to be involved in catalysis. | mitochondrion inner membrane,  single-pass membrane protein, matrix side | 2,03 | 0,001 | # 618252 MITOCHONDRIAL COMPLEX I DEFICIENCY, NUCLEAR TYPE 32; MC1DN32 |
| Q9NX14 | NDUFB11 | NADH dehydrogenase [ubiquinone]  1 beta subcomplex subunit 11, mitochondrial | NDUBB | Accessory subunit of the mitochondrial membrane  respiratory chain NADH dehydrogenase (Complex I), that is believed not to be involved in catalysis. | mitochondrion inner membrane,  single-pass membrane protein. | 2,03 | 0,000 | # 300952 LINEAR SKIN DEFECTS WITH MULTIPLE CONGENITAL ANOMALIES 3; LSDMCA3,  # 301021 MITOCHONDRIAL COMPLEX I DEFICIENCY, NUCLEAR TYPE 30; MC1DN30 |
| P49773 | HINT1 | Histidine triad nucleotide-binding  protein 1 | HINT1 | Exhibits adenosine 5'-monophosphoramidase activity, hydrolyzing purine nucleotide phosphoramidates with a single phosphate group such as adenosine 5'monophosphoramidate (AMP-NH2) to yield AMP and NH2 | cytoplasm, nucleus | 2,00 | 0,001 | # 137200 NEUROMYOTONIA AND AXONAL NEUROPATHY, AUTOSOMAL RECESSIVE; NMAN |
| P13716 | ALAD | Delta-aminolevulinic acid  dehydratase | HEM2 | Catalyzes an early step in the biosynthesis of  tetrapyrroles. | cytosol, extracellular exosome, extracelluar region,  nucleus, ficolin-1-rich granule lumen, secretory granule lumen | 2,00 | 0,005 | # 612740 PORPHYRIA, ACUTE HEPATIC |
| P14868 | DARS | Aspartate--tRNA ligase, cytoplasmic | SYDC | Katalyzes the specific attachment of an amino acid  to its cognate tRNA in a 2 step reaction: the amino acid (AA) is first activated by ATP to form AA-AMP and then transferred to the acceptor end of the tRNA. | cytosol | 0,50 | 0,021 | # 615281 HYPOMYELINATION WITH BRAINSTEM AND SPINAL CORD INVOLVEMENT AND LEG SPASTICITY; HBSL |
| P47897 | QARS | Glutamine-tRNA ligase | SYQ | Glutamine--tRNA ligase. Plays a critical role in brain development | cytosol, cytoplasm | 0,49 | 0,001 | # 615760 MICROCEPHALY, PROGRESSIVE, WITH SEIZURES AND CEREBRAL AND CEREBELLAR ATROPHY; MSCCA |
| Q9UHP9 | SMPX | Small muscular protein | SMPX | Plays a role in the regulatory network  through which muscle cells coordinate their structural and functional states during growth, adaptation, and repair. | nucleus, costamere,  M band, muscle tendon junction | 0,48 | 0,001 | # 300066 DEAFNESS, X-LINKED 4; DFNX4 |
| P05023 | ATP1A1 | Sodium/potassium-transporting  ATPase subunit alpha-1 | AT1A1 | This is the catalytic component of the active enzyme,  which catalyzes the hydrolysis of ATP coupled with the exchange of sodium and potassium ions across the plasma membrane. | basolateral cell membrane,  multi pass membrane protein, sacrolemma, axon, melanosome | 0,47 | 0,022 | # 618036 CHARCOT-MARIE-TOOTH DISEASE, AXONAL, TYPE 2DD; CMT2DD,  # 618314 HYPOMAGNESEMIA, SEIZURES, AND MENTAL RETARDATION 2; HOMGSMR2 |
| Q13698 | CACNA1S | Voltage-dependent L-type  calcium channel subunit alpha-1S | CAC1S | ore-forming, alpha-1S subunit of the voltage-gated calcium  channel that gives rise to L-type calcium currents in skeletal muscle. | T-tubule, multi-pass membrane protein | 0,47 | 0,000 | # 170400 HYPOKALEMIC PERIODIC PARALYSIS, TYPE 1; HOKPP1,  # 601887 MALIGNANT HYPERTHERMIA, SUSCEPTIBILITY TO, 5; MHS5,  # 188580 THYROTOXIC PERIODIC PARALYSIS, SUSCEPTIBILITY TO, 1; TTPP1 |
| P08572 | COL4A2 | Collagen alpha-2(IV)  chain | CO4A2 | Type IV collagen is the major structural component  of glomerular basement membranes (GBM), forming a 'chicken-wire' meshwork together with laminins, proteoglycans and entactin/nidogen. | basement membrane | 0,47 | 0,045 | # 614483 BRAIN SMALL VESSEL DISEASE 2; BSVD2, # 614519 HEMORRHAGE, INTRACEREBRAL, SUSCEPTIBILITY TO; ICH |
| P04792 | HSPB1 | Heat shock protein  beta-1 | HSPB1 | Small heat shock protein which functions as a  molecular chaperone probably maintaining denatured proteins in a folding-competent state | cytoplasm, nucleus, spindle | 0,47 | 0,005 | # 606595 CHARCOT-MARIE-TOOTH DISEASE, AXONAL, TYPE 2F; CMT2F,  # 608634 NEURONOPATHY, DISTAL HEREDITARY MOTOR, TYPE IIB; HMN2B |
| P08708 | RPS17 | 40S ribosomal protein  S17 | RS17 | RNA binding , structural constituent of ribosome, cytoplasmic translation, erythrocyte homeostasis, ribosomal small subunit biogenesis, rRNA processing, translation  translational initiation | cytosol, cytosolic ribosome, cytosolic ribosome, cytosolic small  ribosomal subunit, nucleoplasm, focal adhesion, membrane, ribosome | 0,46 | 0,019 | # 612527 DIAMOND-BLACKFAN ANEMIA 4; DBA4 |
| Q00839 | HNRNPU | Heterogeneous nuclear  ribonucleoprotein U | HNRPU | DNA- and RNA-binding protein involved in several  cellular processes such as nuclear chromatin organization, telomere-length regulation, transcription, mRNA alternative splicing and stability, Xist-mediated transcriptional silencing and mitotic  cell progression. | centrosome, spindle, spindle pole,  cytoplasm, nucleus, nucleus matrix, nucleus speckle, chromosome, kinetochore, midbody, cell surface, cytoplasmic granule | 0,46 | 0,012 | # 617391 DEVELOPMENTAL AND EPILEPTIC ENCEPHALOPATHY 54; DEE54 |
| O14958 | CASQ2 | Calsequestrin-2 | CASQ2 | Calsequestrin is a high-capacity, moderate affinity,  calcium-binding protein and thus acts as an internal calcium store in muscle. | sarcoplasmic reticulum lumen | 0,44 | 0,023 | # 611938 VENTRICULAR TACHYCARDIA, CATECHOLAMINERGIC POLYMORPHIC, 2; CPVT2 |
| P84077 | ARF1 | ADP-ribosylation  factor 1 | ARF1 | GTP-binding protein involved in protein trafficking  among different compartments. | golgi apparatus, trans-Golgi network membrane,  lipid anchor, perinuclear region, synpatosome, postsynaptic density, membrane | 0,43 | 0,031 | # 618185 PERIVENTRICULAR NODULAR HETEROTOPIA 8; PVNH8 |
| P35908 | KRT2 | Keratin, type II cytoskeletal  2 epidermal | K22E | Probably contributes to terminal cornification. | cytoskeleton (intermediate filament, keratin filament), cytosol, extracellular exosome, extracellular space, nucleus, plasma membrane (cornified envelope), membrane | 0,41 | 0,037 | # 146800 ICHTHYOSIS BULLOSA OF SIEMENS; IBS |
| Q10567 | AP1B1 | AP-1 complex  subunit beta-1 | AP1B1 | Subunit of clathrin-associated adaptor protein  complex 1 that plays a role in protein sorting in the late-Golgi/trans-Golgi network (TGN) and/or  endosomes. | golgi apparatus, clathrin-coated vesicle membrane,  peripheral membrane protein, cytoplasmic side | 0,40 | 0,015 | # 242150 KERATITIS-ICHTHYOSIS-DEAFNESS SYNDROME, AUTOSOMAL RECESSIVE; KIDAR |
| P04259 | KRT6B | Keratin, type II  cytoskeletal 6B | K2C6B | There are at least six isoforms of human type II keratin-6 (K6). There are two types of cytoskeletal and microfibrillar keratin, I (acidic) and II (neutral to basic) (40-55 and 56-70 kDa, respectively). | cytoskeleton (keratin filament),  cytosol, extracellular exosome | 0,40 | 0,016 | # 615728 PACHYONYCHIA CONGENITA 4; PC4 |
| P06737 | PYGL | Glycogen phosphorylase,  liver form | PYGL | Allosteric enzyme that catalyzes the rate-limiting  step in glycogen catabolism, the phosphorolytic cleavage of glycogen to produce glucose-1-phosphate, and plays a central role in maintaining cellular and organismal glucose homeostasis. | cytosol | 0,38 | 0,046 | # 232700 GLYCOGEN STORAGE DISEASE VI; GSD6 |
| P13645 | KRT10 | Keratin, type I  cytoskeletal 10 | K1C10 | Plays a role in the establishment  of the epidermal barrier on plantar skin. | extracellular space, cell surface | 0,35 | 0,029 | # 113800 EPIDERMOLYTIC HYPERKERATOSIS; EHK,  # 607602 ICHTHYOSIS, CYCLIC, WITH EPIDERMOLYTIC HYPERKERATOSIS,  # 609165 ICHTHYOSIS WITH CONFETTI; IWC |
| P48668 | KRT6C | Keratin, type II  cytoskeletal 6C | K2C6C | There are at least six isoforms of human type II keratin-6 (K6). There are two types of cytoskeletal and microfibrillar keratin, I (acidic) and II (neutral to basic) (40-55 and 56-70 kDa, respectively). | cytoskeleton (intermediate filament,  keratin filament), cytosol, extracellular exosome | 0,33 | 0,010 | # 615735 PALMOPLANTAR KERATODERMA, NONEPIDERMOLYTIC, FOCAL OR DIFFUSE; PPKNEFD |
| P13647 | KRT5 | Keratin, type II  cytoskeletal 5 | K2C5 | There are two types of cytoskeletal and microfibrillar  keratin: I (acidic; 40-55 kDa) and II (neutral to basic; 56-70 kDa). | cytoskeleton (intermediate filament, keratin filament), cytosol, extracellular  exosome, nucleus, cytoplasm, membrane | 0,29 | 0,006 | # 601001 EPIDERMOLYSIS BULLOSA SIMPLEX 1D, GENERALIZED, INTERMEDIATE OR SEVERE, AUTOSOMAL RECESSIVE; EBS1D,  # 131760 EPIDERMOLYSIS BULLOSA SIMPLEX 1A, GENERALIZED SEVERE; EBS1A, ICD+ # 609352 EPIDERMOLYSIS BULLOSA SIMPLEX 2E, WITH MIGRATORY CIRCINATE ERYTHEMA; EBS2E,  # 131800 EPIDERMOLYSIS BULLOSA SIMPLEX 1C, LOCALIZED; EBS1C,  # 131900 EPIDERMOLYSIS BULLOSA SIMPLEX 1B, GENERALIZED INTERMEDIATE; EBS1B,  # 131960 EPIDERMOLYSIS BULLOSA SIMPLEX 2F, WITH MOTTLED PIGMENTATION; EBS2F,  # 179850 DOWLING-DEGOS DISEASE 1; DDD1 |
| P25189 | MPZ | Myelin protein P0 | MYP0 | Is an adhesion molecule necessary for normal myelination in the peripheral nervous system. | cell membrane, single-pass type I membrane protein,  myelin membrane | 0,28 | 0,023 | # 118200 CHARCOT-MARIE-TOOTH DISEASE, DEMYELINATING, TYPE 1B; CMT1B,  # 607677 CHARCOT-MARIE-TOOTH DISEASE, AXONAL, TYPE 2I; CMT2I,  # 607736 CHARCOT-MARIE-TOOTH DISEASE, AXONAL, TYPE 2J; CMT2J, # 103100 ADIE PUPIL,  # 607791 CHARCOT-MARIE-TOOTH DISEASE, DOMINANT INTERMEDIATE D; CMTDID,   # 145900 HYPERTROPHIC NEUROPATHY OF DEJERINE-SOTTAS,  # 180800 ROUSSY-LEVY HEREDITARY AREFLEXIC DYSTASIA,   # 618184 NEUROPATHY, CONGENITAL HYPOMYELINATING, 2; CHN2 |
| P54136 | RARS | Arginine--tRNA ligase,  cytoplasmic | SYRC | Forms part of a macromolecular complex that  catalyzes the attachment of specific amino acids to cognate tRNAs during protein synthesis | cytoplasm, cytosol | 0,27 | 0,015 | # 616140 LEUKODYSTROPHY, HYPOMYELINATING, 9; HLD9 |
| P61313 | RPL15 | 60S ribosomal  protein L15 | RL15 | cadherin binding, RNA binding, structural constituent of ribosome, cytoplasmic translation, response to ethanol, translation | membrane, lipid-anchor | 0,23 | 0,000 | # 615550 DIAMOND-BLACKFAN ANEMIA 12; DBA12 |
| P04264 | KRT1 | Keratin, type II  cytoskeletal 1 | K2C1 | May regulate the activity of kinases such as PKC  and SRC via binding to integrin beta-1 (ITB1) and the receptor of activated protein C kinase 1 (RACK1). | cell membrane | 0,23 | 0,001 | # 113800 EPIDERMOLYTIC HYPERKERATOSIS; EHK,   # 146590 ICHTHYOSIS HYSTRIX, CURTH-MACKLIN TYPE; IHCM,   # 600962 PALMOPLANTAR KERATODERMA, NONEPIDERMOLYTIC; NEPPK,  # 607602 ICHTHYOSIS, CYCLIC, WITH EPIDERMOLYTIC HYPERKERATOSIS,  # 607654 KERATOSIS PALMOPLANTARIS STRIATA III; PPKS3 |
| P08779 | KRT16 | Keratin, type I cytoskeletal 16 | K1C16 | Epidermis-specific type I keratin that plays a key role  in skin. Acts as a regulator of innate immunity in response to skin barrier breach: required for some inflammatory checkpoint for the skin barrier maintenance. | cytoskeleton, intermediate filament,  cytosol, extracellular exosome, nucleus | 0,20 | 0,004 | # 167200 PACHYONYCHIA CONGENITA 1; PC1,  # 613000 PALMOPLANTAR KERATODERMA, NONEPIDERMOLYTIC, FOCAL 1; FNEPPK1 |
| P35527 | KRT9 | Keratin, type I  cytoskeletal 9 | K1C9 | May serve an important special function either in the  mature palmar and plantar skin tissue or in the morphogenetic program of the formation of these tissues. Plays a role in keratin filament assembly. | cytoskeleton (intermediate filament), cytosol,  extracellular exosome, extracellular space, nucleus, membrane | 0,16 | 0,004 | # 144200 PALMOPLANTAR KERATODERMA, EPIDERMOLYTIC; EPPK |
| Q13618 | CUL3 | Cullin-3 | CUL3 | Core component of multiple cullin-RING-based BCR  (BTB-CUL3-RBX1) E3 ubiquitin-protein ligase complexes which mediate the ubiquitination and subsequent proteasomal degradation of target proteins. BCR complexes and ARIH1 collaborate in tandem to mediate ubiquitination of target proteins. | spindle, centrosome, spindle pole,  cytoplasm, nucleus, golgi apparatus, flagellum | 0,11 | 0,038 | # 614496 PSEUDOHYPOALDOSTERONISM, TYPE IIE; PHA2E |

Green = upregulated proteins, red= downregulated proteins.
